# Red-Fleshed Apple Anthocyanin Extract Reduces Furan Content in Ground Coffee, Maillard Model System, and Not-from-Concentrate Apple Juice

**DOI:** 10.3390/foods10102423

**Published:** 2021-10-13

**Authors:** Bin Wang, Shenghui Jiang, Yanbo Wang, Jihua Xu, Meng Xu, Xiaohong Sun, Jun Zhu, Yugang Zhang

**Affiliations:** 1 Engineering Laboratory of Genetic Improvement of Horticultural Crops of Shandong Province, Qingdao Agricultural University, Qingdao 266109, China; WB765198@163.com (B.W.); mingsun9887@163.com (X.S.); 2 College of Horticulture, Qingdao Agricultural University, Qingdao 266109, China; jsh2862317@163.com (S.J.); 15820054339@163.com (Y.W.); mengxu0214@163.com (M.X.); 3 College of Life Sciences, Qingdao Agricultural University, Qingdao 266109, China; xujihua@qau.edu.cn

**Keywords:** furan, red-fleshed apple, anthocyanin, Maillard reaction, NFC apple juice

## Abstract

Furan is a volatile and carcinogenic heterocyclic chemical compound that occurs in a wide range of thermally processed food. It can be induced during food-preparing processes by high temperatures and UV-C light. In the present study, the degradation of furan content in ground coffee, Maillard model system, and not-from-concentrate (NFC) apple juice by red-fleshed apple anthocyanin extract (RAAE) was studied. The results demonstrated that RAAEs had different degrees of degradation of furan content in coffee powder, and the RAAE from ‘XJ3’ had the most significant effect, with a reduction rate of up to 20%. Moreover, by adding RAAE to the Maillard model system, we found the amounts of furan were significantly reduced. At the same time, RAAE from ‘XJ3’ could observably reduce the content of furan in pasteurized NFC juice, with ‘Fuli’ NFC juice furan content decreasing the most, which was 68%. Taken together, our study demonstrated that the use of RAAE could be a feasible way to reduce furan content in ground coffee, Maillard model system, and NFC apple juice.

## 1. Introduction

Furan, one of the simplest oxygen-containing pentacyclic heterocompounds, has a low boiling point. It is classified by International Agency for Research on Cancer (IARC) as possibly carcinogenic to humans. Animal evidence suggests that low doses of furans are strongly carcinogenic [1]. Studies have revealed that residual furan in the body accumulates mainly in the liver and to a lesser extent in the kidneys and colon [2]. As furan in food has become a public health concern, it is essential to understand how furans are formed during the heat treatment of foods and to control their production. Therefore, in recent years, many scholars have widely started to study furans and their derivatives in heat-processed foods [3]. Studies have shown that the thermal degradation of sugars is a pathway for furan production [4]. Previous studies had found that furan was produced during the simulation of the Maillard reaction and pointed out several pathways for furan production by thermal degradation of sugars: first, sugars go through a cleavage process to form butyralose, which then forms furans through cyclization and dehydration; second, in the presence of amino acids, sugars undergo a Maillard reaction with amino acids to produce intermediates and then undergo a series of reactions to form butyralose and its derivatives, which form furans through cyclization and dehydration [5]. In addition to heat treatment being able to induce furan formation in foods, nonthermal ultraviolet C (UV-C) light treatment can also lead to furan formation in various foods, such as fruit juices and sugar solutions [6]. Studies had indicated that fructose, the main component in fruit juices, forms furans during UV-C light treatment, although the mechanism of formation has not been determined [7].

Food ingredients generally do not contain furans, but sugars, amino acids, and ascorbic acid in raw materials are subjected to a Maillard reaction or oxidative degradation during thermal processing or storage, and a large number of furan compounds are formed [8]. Maillard reaction is a common chemical reaction that occurs during the heat treatment of food processing and contributes to the sensory quality of food. Coffee is a common drink in people’s ordinary life, and there have been a large number of reports on the health benefits and risks of coffee drinking [9]. While furan levels in raw coffee beans are extremely low and almost undetectable, after roasting, the furan content reaches as high as 6 mg/kg in coffee beans [10]. Thus, furan is mainly produced during the roasting of carbohydrates and amino acids, which are rich in raw coffee beans [5].

Since furans are highly volatile and susceptible to interference from other substances in the food and the external environment during the content quantification [11], it is crucial to maintain the stability of the testing environment for the determination of furan content. According to current technology, there are three main methods for the determination of furans in food: headspace injection-gas chromatography-mass spectrometry [12], solid-phase microextraction-gas chromatography-mass spectrometry [13] and headspace solid-phase microextraction-gas chromatography-flame ionization [14]. Headspace gas chromatography-mass spectrometry is commonly used for furan detection [11].

There are three main traditional ways to reduce furan generation: first, control the formation of key intermediates; second, the inhibition of key steps in the Maillard reaction; third, the addition of antioxidants, which transform the reaction toward the formation of other small molecules. Studies have shown that heating temperature and time are closely related to the amount of furan production in foods [15]. It has been demonstrated that in apple juice processing, furan production can be mitigated to some extent by replacing the traditional pasteurization method with ultrahigh-pressure homogenization [16]. However, most of these methods may affect the sensory characteristics of foods, including texture, color, and taste.

In recent years, some natural products have attracted attention for their excellent antioxidant capacity, such as caffeic acid, tea polyphenols, melatonin, and chondroitin, and all were shown to significantly reduce furan content [17,18,19]. Previous studies have investigated the effects of dicarbonyl-trapping agents (epigallocatechin, epigallocatechin gallate, and catechin), water-soluble antioxidants (caffeic acid, ferulic acid, and chlorogenic acid), fat-soluble antioxidants (α-tocopherol, BHT, and β-carotene), and reducing agents (glutathione and sodium sulfite) on furan formation in the canned coffee system [20].

Red-fleshed apples are rich in anthocyanins, which are the main color-presenting substances in the plant. Anthocyanins are rare in their free state under natural conditions and exist mainly in the form of glycosides. Anthocyanins are highly hydrophilic, which makes them play a protective role in various pathophysiological conditions [21], such as improving the subjective symptoms of visual fatigue after work [22], preventing of cardiovascular diseases [23], and protecting the liver from damage [24]. The anthocyanins in the juice of blueberry showed strong antioxidant activity to superoxide anion free radicals [25]. In addition, in our previous studies, it was found that red-fleshed apples have significant antioxidant capacity in vitro [26,27]. However, studies on the reduction of furan content in the process of coffee, simulated furan solutions, and not-from-concentrated (NFC) juices by anthocyanins are very limited, and anthocyanin extracts are likely to be used as inhibitors for furan formation.

In this study, the headspace conditions were optimized and then coupled to a gas chromatograph to reduce the instrumentation costs. The aim of this study was to investigate the effect of antioxidants on furan content in ground coffee, Maillard model system, and NFC apple juice. Red-fleshed apple anthocyanin extract was chosen as an antioxidant because of its high free-radical scavenging ability, and previous studies demonstrated that RAAE has strong antioxidant properties both in vitro and in vivo [26,28]. We speculated that RAAE might have a decreasing effect on furan content. RAAE is a new choice of antioxidant with high safety, low production cost, and simple operation.

## 2. Materials and Methods

### 2.1. Reagents and Materials

Furan (99%) was purchased from Solarbio Technology (Beijing, China). Chromatographically pure phosphoric acid, analytically pure anhydrous ethanol and methanol were obtained from Yongda Chemical Reagent Company (Tianjin, China). Analytical grade glucose and alanine were purchased from Solarbio Technology Co. L-ascorbic acid and Cyanidin 3-O-galactopyranoside were obtained from Sigma-Aldrich (St. Louis, MO, USA). In this work, the material of RAAE and NFC apple juice were the new variety and hybrid offspring bred by our team (Tables S1 and S2). The apple varieties (strains) were all planted in Jiaozhou Modern Agricultural Science and Technology Demonstration Park of Qingdao Agricultural University. When the fruits reach maturity, they were harvested and placed in cold storage (4 °C) for precooling on the same day and were pressed for juice on the next day. The fruits quality used for this study were all consistent, without any disease, pests, or mechanical damages.

### 2.2. HS-GC-MS Standard Curve External Standard Method Was Used to Detect and Analyze the Furan Content in Coffee

A standard stock solution of 1 mg/mL furan, a standard working solution of 1 μg/mL furan, and a standard series of solutions with furan contents of 20, 100, 400, 600, and 800 ng/mL, respectively, were configured for the determination of furan content in the analytical coffee solution. The limit of detection (LOD, S/N = 3) for the method was 1 ng/mL, and the limit of quantity (LOQ) was 3.3 ng/mL. The recovery rate was 96.3% with a low RSD of 3.1%. Solid-phase microextraction-gas chromatography-mass spectrometry was used for the determination of furan in the Maillard model system and NFC apple juice.

Furan standard stock solution and working solution were well prepared, and standard solutions of 0.05, 0.1, 0.5, 1, 5, and 10 ng/mL were used to detect furan in Maillard model system and NFC apple juice, respectively. The limit of detection (LOQ, S/N = 10) for the method was 0.006 ng/mL, and the limit of quantity (LOQ) was 0.025 ng/mL. The recovery rate was 97.4% with a low RSD of 2.7%.

Headspace injection conditions: equilibrium temperature was 55 °C; equilibrium time was 30 min; quantification ring temperature was 100 °C; transfer line temperature was 110 °C; bottle pressurization time was 0.5 min; sample bottle pressure was 103.4 kPa (15 psi); filling time of quantification ring was 0.5 min; quantification ring equilibrium time was 1 min; injection time was 1 min; and the GC cycle time was 30 min. Chromatographic column: Agilent HP-PLOT/Q quartz capillary column (30 m × 0.32 mm × 20 μm); inlet temperature: 200 °C; interface temperature: 230 °C; ramp-up procedure: the starting temperature was 50 °C, held for 1 min and then ramped up to 230 °C at a ramp-up rate of 10 °C/min, held for 10 min; carrier gas: high purity helium gas. The flow rate was 1.2 mL/min. Ion source: high-sensitivity electron bombardment (EI) ion source with electron energy of 70 eV; ion source temperature: 230 °C; quadrupole I and II temperature: 200 °C; scanning method: selected ion monitoring (MSI SIM) was used to detect furan molecular ions m/z 68. Since the furan content varies greatly in different systems, two furan standard curves were plotted in order to more accurately detect the furan content in different systems. Standard curve method I: furan content was linear in the range of 20–800 ng/mL with the linear equation Y = 112,623X – 142,888 and the correlation coefficient of 0.9996. This was used to detect the furan content in coffee in this experiment (Supplementary Materials Figure S1A). In which y is the concentration of furan in the sample and x is the peak area, x ranges between 20 and 800 ng/mL. The Standard curve II was Y = 25,021X − 1258.7 with a correlation coefficient of 0.9993. This was used to detect furan in the furan precursor model solution and NFC apple juice in this experiment (Figure S1B). In which y is the concentration of furan in the sample and x is the peak area, x ranges between 0.05 and 10 ng/mL.

### 2.3. Extraction and Purification by Adsorption Chromatography

**Figure 1:**
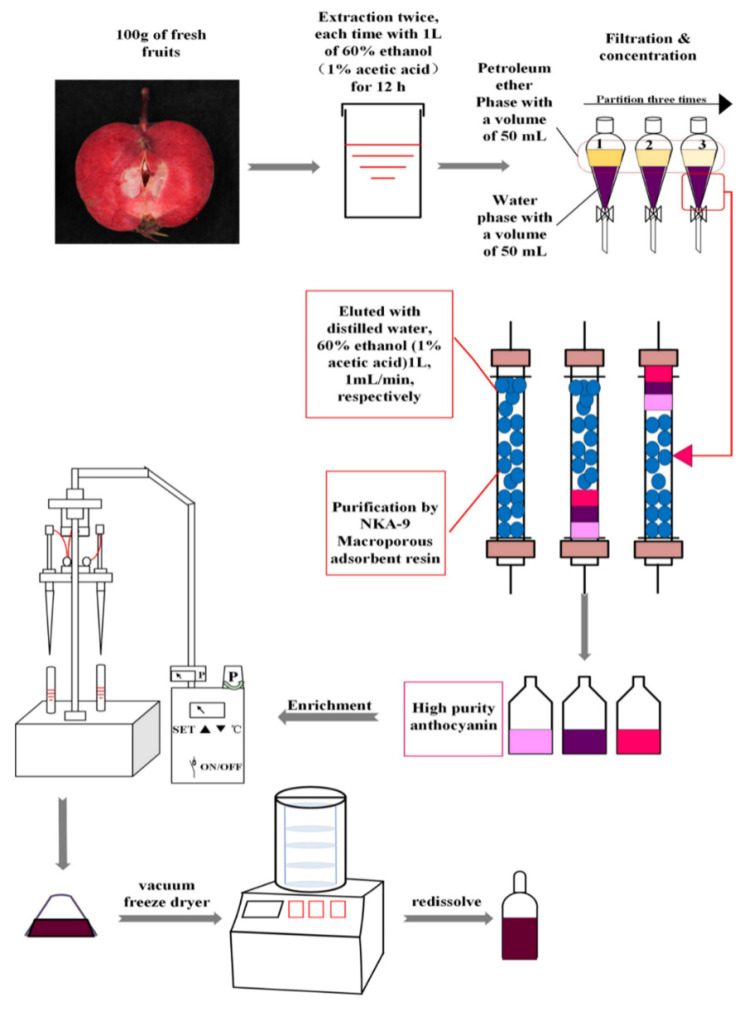
The model diagram of preparation of RAAE.

A schematic of the proposed method for obtaining anthocyanin mixtures is illustrated in Figure 1. The red-fleshed apples were washed and quickly sliced thinly, divided in tin foil, and then snap-frozen in liquid nitrogen. First, 100 g of red-fleshed apple was homogenized in a blender and extracted with 1 L of 0.1% acetic acidified 60% (*v*/*v*) ethanol for 12 h in the dark. After the extracts were filtered, the residue was dissolved with 1 L of 0.1% acetic acidified 60% (*v*/*v*) ethanol and extracted for 24 h using the same conditions previously described. The clear liquid from two extractions was mixed and concentrated to a volume of 50 mL using a nitrogen blowing instrument (NBI) at temperatures not exceeding 40 °C. The resulting aqueous solution (50 mL) was then partitioned sequentially with petroleum ether (PE, 50 mL) three times. Due to the slight solubility of water and PE, the volume of water phase would decrease in each step; thus, a total of about 25 mL aqueous solution was collected and then kept at 4 °C in the dark. The aqueous phase was loaded onto a column (NKA-9, 2.6 cm × 50 cm) of cation-exchange resin (Macroporous adsorption resin; particle size: 0.3–1.2 mm, wet). The column was washed with 2 L of deionized water (0.01% acetic acid) at a flow rate of 1 mL/min to remove the majority of sugars, and acids, and then, elution of anthocyanins was performed using 1 L of 60% ethanol at 1 mL/min. Finally, the eluate was concentrated using a NBI at temperatures not exceeding 40 °C, and the resulting solution was freeze-dried. RAAE was stored at low temperature (−80 °C) and protected from light. The red apple varieties (strains) selected in this study were Xinjiang No.1 (XJ1), Xinjiang No.2 (XJ2), Xinjiang No.3 (XJ3), Xinjiang No.5 (XJ5), Xinjiang No. P5 (XJP5), Xinjiang No. P7 (XJP7), Hongxun No.1 (HX1), Super Red and Red Love.

The coffee samples, including instant coffees and ground coffees, were obtained from a local store in Qingdao, China. For ground coffee samples, the ground coffee beans were purchased from the local store, and brewing was conducted in lab at the time of testing. Here, 1.0 g of ground coffee was rapidly added to each sample and sealed with a buna-PTFE septum and screw-cap. Afterward, the coffee was brewed according to the manufacturer’s instructions or recommendations, stored at 4 °C, and prepared for analysis. Then, 1 g of RAAE was added to each vial using a gas-tight microsyringe and sealed.

### 2.4. UV-C Treatment of the Maillard Model System

UV-C treatment was performed by using a model LPS-425-40 Mineralight lamp (Langpu Optoelectronics Technology Co., Ltd., Guangzhou, China) at 253.7 nm. To prepare the Maillard model system, 1 mmol glucose and alanine were placed in a quartz detachable tube with an internal thickness of 2.0 mm, and ultrapure water was added to fix the weight to 7 g. Then, during UV-C treatment, the quartz cuvette was set directly beneath the UV lamp, and the sample was stirred constantly to achieve even exposure. All samples were stored at 4 °C prior to treatment. The distance between the UV lamp and the cuvette could be adjusted according to the required UV-C intensity. The distance was fixed at 8.0 cm to attain 5.0 mW/cm^2^. UV-C fluences (5, 7, 9, 11, and 15 J/cm^2^) were achieved by setting the different exposure time. The Maillard model system solution was quickly added to the 20 mL screw cap glass headspace vial and sealed with a NITrile PTFE diaphragm and screw cap. After being treated with UV-C and stored at 4 °C for analysis, 1 g RAAE was then added to each vial using a gas-tight microsyringe and sealed.

### 2.5. Preparation of NFC Apple Juice

The apple varieties (strains) selected in this study were Fuji, Fuli, Fuxing, 2010-W13-11Z-N9, 2008-W18-N16, 2010-W14-11Z-N9, 2010-W11-5Z-N4, 2010-W16-13Z-N7, 2010-W10-3Z-N2, 2009-E7-N44, 2010-W5-N2, and 2010-W15-12Z-N1. Apple fruits with consistent quality and weight were selected and washed to remove dust and impurities and then cut into pieces for pressing treatment, and the pressed solution was filtered to remove slag. The filtrate was pasteurized, and after sterilization, it was filled and sealed for storage.

### 2.6. Sensory Assessment

A sensory assessment team conducted sensory evaluation on the coffee and NFC apple juice. Sensory evaluation of coffee was performed as previously reported with a slight modification [29]. Sensory evaluation of NFC apple juice was carried out as previous description with a slight modification [30,31].

### 2.7. Statistical Analysis

Values were expressed as means ± standard deviation (SD). Each series of experiments was repeated three times. The groups in the data were analyzed by one-way analysis of variance (ANOVA), and *p* < 0.05 was considered statistically significant.

## 3. Results and Discussion

### 3.1. Effects of Different RAAE on the Inhibition of Furan in Ground Coffee

**Figure 2:**
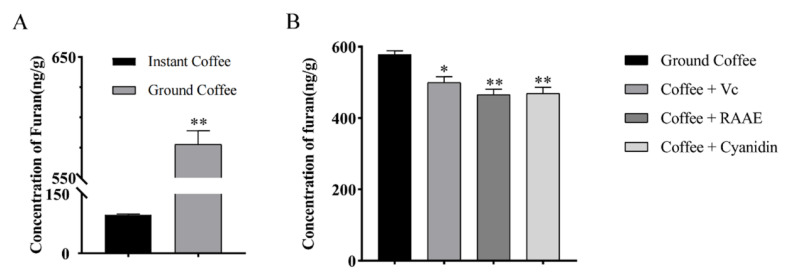
(**A**) Furan content in commercially available instant coffee and ground coffee and (**B**) the reduction effect of different antioxidants on the furan content in coffee. * means *p* < 0.05, and ** means *p* < 0.01.

In this study, furan concentrations in commercially available instant coffee and ground coffee beans were first measured by HS-GC-MS. It was found that (Figure 2A) the furan concentration in the ground coffee was significantly higher than that in the commercially available instant coffee. The furan concentration was 5.96 times higher than that in the instant coffee. Based on the results, we selected ground coffee as the material to fully represent the variation of furan concentration. It has been reported that antioxidants such as ascorbic acid and gallic acid have a decreasing effect on furan concentration [6]. According to our previous study, whatever in vitro and in vivo, RAAE were beneficial in combating oxidation. Therefore, we hypothesized that RAAE as a natural antioxidant may significantly reduce the content of furan in coffee and other solutions. We used airtight microsyringes to add different antioxidants of the same concentration (10 μg/mL) to the same amount of coffee solution and sealed it. In addition, 1.25 µg of antioxidant was added to 1 mL of coffee solution, and the result is shown in Figure 2B. Compared with the ground coffee group, the furan concentration in the coffee solution after the addition of the ascorbic acid showed a significant trend of 485 ng/g, and the reduction rate was 16%. When RAAE and Cyanidin-3-O-glucoside standard (Coffee + Cyanidin) with the same concentration were used to replace ascorbic acid, the concentration of furan in the sample showed a more significant decreasing trend, which was 460 and 460 ng/g, respectively. The reduction rates were 21% and 20%, respectively. Therefore, it could be preliminarily concluded that RAAE could reduce the content of furan in coffee, and the scavenging effect was stronger than that of ascorbic acid with the same concentration.

**Figure 3:**
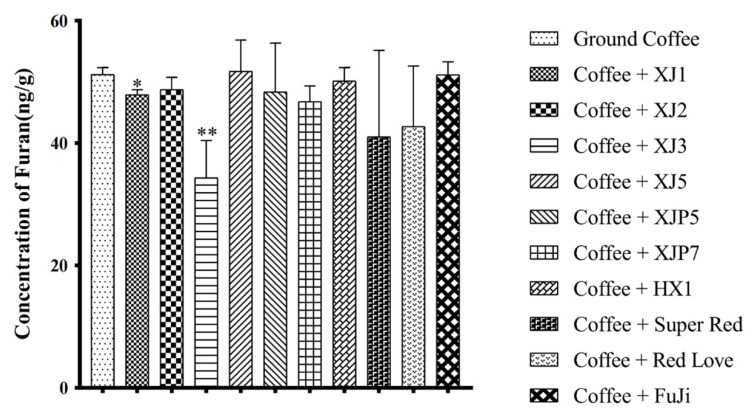
The reduction effect of different varieties of RAAE on furan content in ground coffee. * means *p* < 0.05, and ** means *p* < 0.01.

In order to distinguish the reduction rate of furan content in different red-fleshed apple varieties, 10 different types of RAAEs were added to the same coffee solution. Before that, we measured the furan concentration of the ground coffee group, where the furan concentration was 51 ng/mL. Among the nine selected RAAEs, two significantly reduced the contents of furan, while the other seven species reduced the contents of furan to some extent, but the difference was not significant (Figure 3). From the results, it was obvious that RAAE of ‘XJ3’ had the most significant reduction effect, and the reduction rate was 33%. The addition of RAAE significantly reduced the content of furan in ground coffee.

The formation of furans in coffee solution is known to be closely related to the products of the Maillard reaction, such as butyralose and its derivatives, forming furan by cyclization and dehydration [5]. Anthocyanins have strong antioxidant properties and the free-radical scavenging ability to inhibit the Maillard reaction and fat oxidation reaction; therefore, they can be added to coffee solutions to inhibit furan. The contents of total phenols and anthocyanins in ‘XJ3’ were the highest among the 10 candidate apple varieties (Table S1). Previous studies have shown that there was a significant correlation between phenolic content and antioxidant capacity [32]. Bi found that tea polyphenols had excellent antioxidant capacity and free-radical scavenging ability and can inhibit the oxidation reaction of Maillard and fat and can inhibit the generation of furan in the canned coffee model [18]. The antioxidant activity of RAAE was found positively correlated with the contents of total phenols and anthocyanins in the fruit in our research. Therefore, we concluded that the reduction of furan content in coffee by RAAE was closely related to its antioxidant capacity.

### 3.2. Effects of Different Concentrations of ‘XJ3’ RAAE on the Inhibition of Furan in Coffee

**Figure 4:**
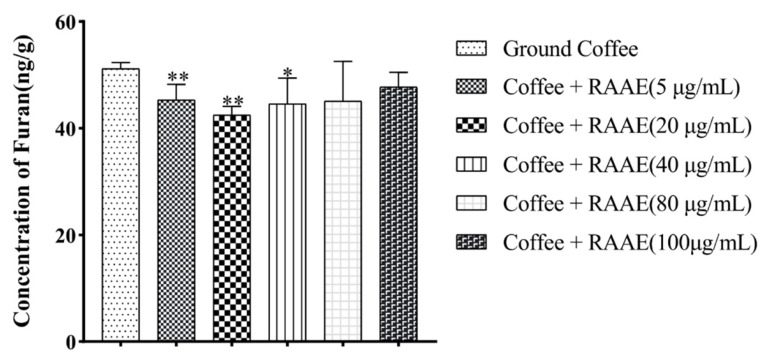
The reduction effect of different concentrations of ‘XJ3′ RAAE on the furan content in coffee. * means *p* < 0.05, and ** means *p* < 0.01.

Anthocyanins are effective antioxidants, and they effectively burst free radicals and terminate the chain reaction of oxidative damage. Among the anthocyanin-rich plants, it was recognized that cyanidin-3-O-galactopyranoside accounted for a large proportion, usually more than 50% of the anthocyanin content [26]. Thus, we set up an RAAE concentration gradient using ‘XJ3’ RAAE in order to find out the concentration of anthocyanin with the best effect on furan inhibition in coffee. Five concentration gradients (5, 20, 40, 80, and 100 μg/mL) were used, with the furan content 44, 42, 45, 45, and 48 ng/mL, respectively. The results indicated 20 μg/mL of anthocyanins had the most significant reduction effect on furan in coffee, and the reduction rate was 17%. While 5, 40, 80, and 100 μg/mL of anthocyanins extracts also had a significant reduction effect, the reduction rates were 15%, 13%, 12%, and 7%, respectively (Figure 4). In addition, we found there was a nonlinear relationship between anthocyanin concentration and reduction rate.

### 3.3. Effects of RAAE on Furan Concentration in Maillard Model System

**Figure 5:**
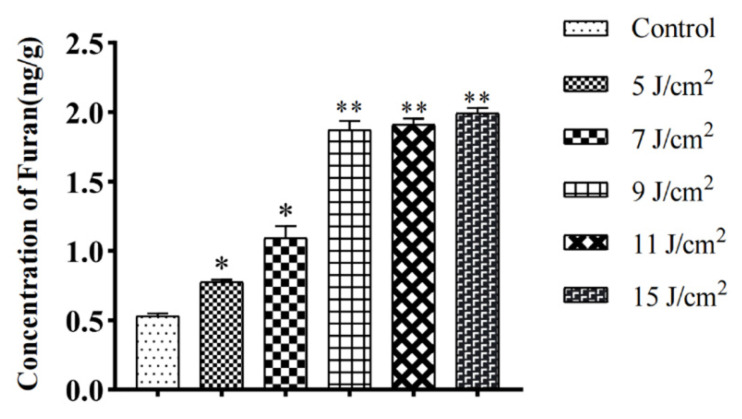
Furan concentration after UV-C treatment of glucose-alanine simulated solution. * means *p* < 0.05, and ** means *p* < 0.01.

**Figure 6:**
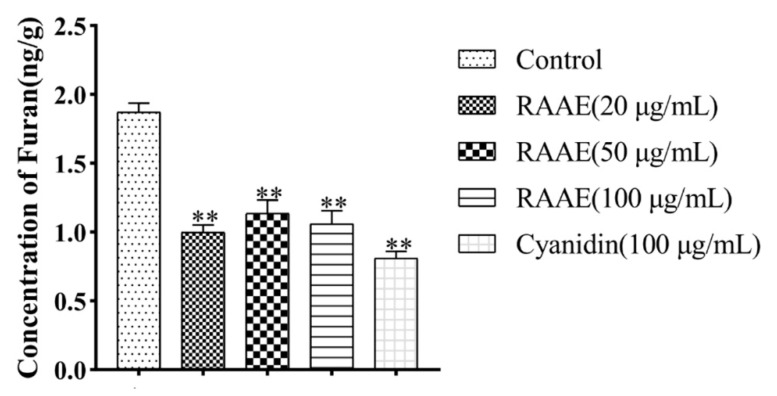
The reduction effect of RAAE on furan content in simulated solution in the Maillard model system. ** means *p* < 0.01 vs. control group.

Here, we detected the different rates of furan formation in the Maillard model system following different UV-C treatments (Figure 5). However, with the increase in time, when the exposure time of UV-C reaches a certain degree, the content of furan in the system does not increase significantly. The results showed that, compared with the control group, the furan concentration in the Maillard model system increased to 0.78 ng/mL. Under UV-C irradiation at 7 J/cm^2^, the concentration of furan in the solution was 1.1 ng/mL, and the irradiation dose of furan in the Maillard model system at 9 J/cm^2^ was 1.9 ng/mL. While the concentration of furan in the solution did not increase significantly after higher radiation doses, 11 and 15 J/cm^2^ irradiation resulted in furan concentrations of 1.9 and 2.0 ng/mL. Since the increase in furan content in 11 and 15 J/cm^2^ is not significant compared with 9 J/cm^2^, we chose 9 J/cm^2^ for the Maillard model system UV irradiation. Subsequently, in order to further verify the reduction effect of RAAE as a natural antioxidant on furan content in Maillard model system, we treated RAAE with different concentrations (20, 50, and 100 μg/mL). The furan content in the solution with RAAE was significantly reduced to 1.0 ng/mL, and the furan concentration in the cyanidin-3-O-galactopyranoside (cyanidin) group was 1.0 ng/mL. The reduction effect of RAAE on furan in the Maillard model system was extremely significant, and there was no significant difference compared with the cyanidin group (Figure 6).

Furan formation in sugar solutions and apple cider during UV-C treatment was first reported by Fan and Geveke [7]. Later, Bule confirmed the formation of furan during UV-C treatment in high-fructose corn syrup (HFCS) solutions and simulated juices, apple juice, and apple cider [33]. Blue also found that the addition of ascorbic acid (AA) to these solutions inhibited the synthesis of furan, and the absorbance of the solution indicated the degree of effectiveness of the inhibition of furan [33]. In this study, considering a photoreaction mechanism, we hypothesized that furan formation was induced by free radicals during UV-C treatment, and the content of furan could be reduced by adding antioxidants. Our results supported this hypothesis and provided potential solutions to control furan in UV-C treated solutions during production.

### 3.4. Effect of RAAE on Furan in NFC Apple Juice

In recent years, due to the gradual acceptance and pursuit of nonconcentrated reduced juice, the proportion of NFC juice in the juice market has increased significantly. NFC juice contains fructose, glucose, amino acids, and other furan synthesis precursors.

**Figure 7:**
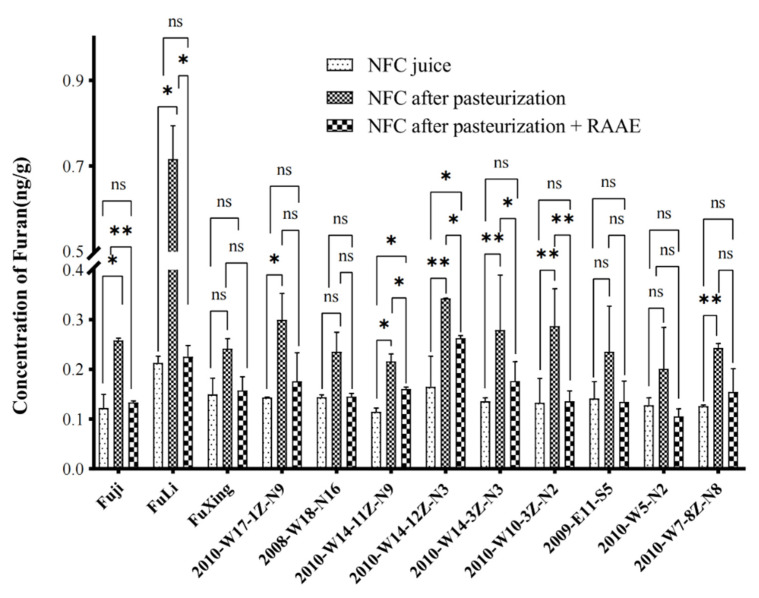
The furan content of NFC apple juice after pasteurization and the reducing effect of RAAE on furan afterward. * means *p* < 0.05, ** means *p* < 0.01, and ns means no significant difference.

In this study, 11 apple varieties (strains) with high sugar and high acid were selected by using the existing apple variety resources in our laboratory. Fuji was used as the control variety, which is recognized as excellent fresh food variety in the world. In total, seven traits of fruit and juice were measured and evaluated (Table S2). The fruit quality index level of 11 apple varieties (strains) was 97–240 g, the average fruit weight was 144 g, and the fruit size was moderate. The content of titratable acid in seven high-acid apple varieties (strains) ranged from 0.61% to 0.68%, which was higher than the requirement of 0.60%. The soluble solids content of four high sugar apple varieties (strains) ranged from 13.4% to 16.7%. Limacher reported that a large amount of furan was produced in the solution of glucose, fructose, arabinose, and erythritosaccharide under dry heat, with arabinose producing the most amount of furan [34]. Ascorbic acid is easily oxidized to dehydroascorbic acid, which is then hydrolyzed to 2-diketogulonic acid, which is then decomposed with α-dicarbonyl to yield butylaltose and furan. However, part of ascorbic acid could not be oxidized to 2-deoxyuronic acid in the condition of anaerobic decomposition. After hydrolysis and β-elimination, 3-deoxypentantose was formed by decarboxylic reaction, and 2-deoxybutylaldose was formed by cleavage according to the α-dicarbonyl of ribose, which could directly form furan. Therefore, furan can be produced under either anoxic or oxygen-rich conditions [5]. In this study, all the selected apple strains meet the requirements of juice-making varieties, but the risk is that the processing of NFC juice with heating could increase the content of furan. In addition, this study verified that the concentration of furan in NFC juice was significantly enhanced after pasteurization (Figure 7). Among them, the content of furan in the NFC juice of eight varieties increased significantly (*p* < 0.05) and that of ‘Fuli’ variety was the highest, up to 0.7 ng/g. Although there was no significant difference in furan content among the other four NFC juices, all of them increased to a certain extent. Rasim studied the inhibitory effects of seven polyphenols and three plant extracts of furan compounds in the Maillard reaction simulation system. The results showed that all phenolic substances and plant extracts except olive polyphenols could reduce the content of furan compounds in the system [17]. In addition, in this study, we preliminarily concluded that RAAE did have a certain reduction effect on furan. Therefore, we speculated that RAAE had a positive effect on reducing furan concentration in NFC apple juice.

The furan concentrations in NFC apple juice were all significantly reduced after the addition of the same content of RAAEs from all strains. Among them, the content of furan in NFC juice of six varieties decreased significantly (*p* < 0.05), while the difference of furan content in the other six varieties of NFC juices was not significant, but all of them decreased to different degrees. The greatest reduction in the concentration of NFC juice furan of ‘Fuli’ was 68%. Tea polyphenols can inhibit furan increases in canned coffee by inhibiting the Maillard reaction, Strecker degradation, decomposition of sulfur-containing amino acid and hydroxyl amino acid, and the fat oxidation [18]. Our results have demonstrated that RAAE can also reduce the content of furan in the Maillard reaction. Adding RAAE at a certain concentration can effectively reduce the furan content. Studies have shown that tea polyphenols can effectively reduce the furan content in canned coffee because tea polyphenols block key steps in the Maillard reaction. Tea polyphenols and anthocyanins have the strong ability of scavenging free radicals and are good antioxidants. Therefore, we speculate that anthocyanins have a similar function or that redirecting the reaction later favors the formation of other small molecules. NFC apple juice is rich in fructose, and fructose is one of the effective furan precursors [34]. Therefore, the reduction of volatile aromatic components in RAAE may be due to the quenching of intermediates formed by the reaction of catechins with sugar fragments, which is in good agreement with the reported results [20]. Considering additional RAAE may affect the color and taste of the drink, we conducted a sensory assessment on both coffee and NFC apple juice after adding RAAE. In the coffee solution, since the color of coffee was brown, there was almost no color changes after adding RAAE. In terms of taste, the low concentrations of RAAE had no effects on the taste of coffee, but high concentrations of RAAE could destroy the taste of coffee (Table S3). In NFC apple juice, adding RAAE did not significantly change the taste of the juice, but in a few apple varieties, the juice became darker (Table S4).

## 4. Conclusions

In this study, the effects of different varieties and concentrations of RAAEs on furan were studied using ground coffee, Maillard model system, and NFC apple juice as experiment systems. Firstly, we studied the effects of different varieties of RAAE and different concentrations of RAAE on the content of furan in coffee. We found that RAAEs could be used to reduce the content of furan in the Maillard model system during UV treatment. The 10 screened high-sugar, high-acid NFC apple juices showed a significantly increased furan content after pasteurization, and the addition of RAAE significantly reduced the furan concentration in pasteurized NFC apple juices.

## Supplementary Materials

The following are available online at https://www.mdpi.com/article/10.3390/foods10102423/s1, Figure S1: (A) Standard curve of furan in the concentration range 20–800 ng/mL; (B) standard curve of furan in the concentration range 0.1–10 ng/mL. Figure S2. Appearance of fruits and juices of different apple varieties (strains). Table S1. Physiological indexes of RAAEs. Table S2. Characteristics detection and quality evaluation of fruit and juice for processing juice in apple varieties (strains) selected in this study. Table S3. Sensory assessment of ground coffee after adding RAAE. Table S4. Sensory assessment of NFC apple juice after adding RAAE.
