# Supplementary material for: Red-Fleshed Apple Anthocyanin Extract Reduces Furan Content in Ground Coffee, Maillard Model System, and Not-from-Concentrate Apple Juice
Source: Foods. 2021 Oct 13;10(10):2423. doi: 10.3390/foods10102423 (PMC8535902; doi:10.3390/foods10102423)
Supplement: Supplementary file 1 [file foods-10-02423-s001.zip › foods-1386660-supplementary/Supplementary Materials/Supplementary Materials- Table S1.pdf]

Table S1. Physiological indexes of RAAEs.

No.	Variety (Strain)	Fruit Weight (g)	Anthocyanin (mg/kg)	Total Phenols (mg/kg)
1	XJ1	15.30	455.00	4983.00
2	XJ2	19.45	146.13	2451.43
3	XJ3	23.42	476.18	6135.52
4	XJ5	17.49	125.31	1893.00
5	XJP5	18.73	364.94	4979.88
6	XJP7	17.82	350.47	4618.52
7	HX1	20.61	249.44	2915.79
8	Super Red	43.59	238.19	5162.87
9	Red Love	125.21	132.67	5468.42
10	Fuji	231.45	12.80	221.24
