# Supplementary material for: Red-Fleshed Apple Anthocyanin Extract Reduces Furan Content in Ground Coffee, Maillard Model System, and Not-from-Concentrate Apple Juice
Source: Foods. 2021 Oct 13;10(10):2423. doi: 10.3390/foods10102423 (PMC8535902; doi:10.3390/foods10102423)
Supplement: Supplementary file 1 [file foods-10-02423-s001.zip › foods-1386660-supplementary/Supplementary Materials/Supplementary Materials- Table S2.pdf]

Table S2. Characteristics detection and quality evaluation of fruit and juice for processing juice in apple varieties (strains) selected in this study.

No.	Variety (Strain)	Fruit Weight (g)	pH	Juice Yield (%)	TA (%)	TSS (°Brix)	Total Phenols (mg/L)	Color Value (L)
1	Fuji	155.73	3.90	62.08	0.377	11.40	1730.74	30.95
2	Fuli	239.80	4.43	67.94	0.280	16.70	2713.82	30.74
3	Fuxing	119.70	4.26	64.15	0.380	13.40	2020.21	33.62
4	2010-W17-1Z-N9	100.55	3.96	62.96	0.330	14.50	1112.32	28.52
5	2008-W18-N16	113.73	3.53	66.19	0.270	14.60	2862.32	30.02
6	2010-W14-11Z-N9	131.30	3.45	52.75	0.620	10.40	2605.74	30.78
7	2010-W14-12Z-N3	104.23	4.38	50.38	0.684	14.03	2303.11	34.69
8	2010-W14-3Z-N3	97.43	3.66	64.11	0.640	11.90	1566.26	31.51
9	2010-W10-3Z-N2	180.73	3.54	70.59	0.680	12.10	2980.74	33.44
10	2009-E11-S5	138.62	4.03	53.21	0.610	12.47	3243.89	36.99
11	2010-W5-N2	218.20	3.56	46.06	0.620	10.20	1586.00	30.19
12	2010-W7-8Z-N8	133.10	3.57	54.33	0.640	11.00	3901.79	37.40
