# Supplementary material for: Red-Fleshed Apple Anthocyanin Extract Reduces Furan Content in Ground Coffee, Maillard Model System, and Not-from-Concentrate Apple Juice
Source: Foods. 2021 Oct 13;10(10):2423. doi: 10.3390/foods10102423 (PMC8535902; doi:10.3390/foods10102423)
Supplement: Supplementary file 1 [file foods-10-02423-s001.zip › foods-1386660-supplementary/Supplementary Materials/Supplementary Materials- Table S3.pdf]

Table S3. Sensory assessment of ground coffee after adding RAAE.

Group	Color	Aroma	Abundance	Astringency	Aftertaste	Global Impression
Ground Coffee	brown	flowery	mellow	soft	rich	supple
Coffee + XJ1 (10 μg/mL)	brown	flowery	moderate	micro astringent	rich	supple
Coffee + XJ2 (10 μg/mL)	brown	flowery	mellow	soft	rich	supple
Coffee + XJ3 (5 μg/mL)	brown	flowery	mellow	soft	rich	full
Coffee + XJ3 (10 μg/mL)	brown	flowery	mellow	soft	rich	full
Coffee + XJ3 (20 μg/mL)	brown	flowery	mellow	soft	rich	supple
Coffee + XJ3 (40 μg/mL)	brown	flowery	mellow	soft	rich	supple
Coffee + XJ3 (80 μg/mL)	brown	spicery	moderate	soft	rich	supple
Coffee + XJ3 (100 μg/mL)	brown	spicery	moderate	micro astringent	rich	supple
Coffee + XJ5 (10 μg/mL)	brown	flowery	mellow	soft	rich	supple
Coffee + XJP5 (10 μg/mL)	brown	flowery	mellow	soft	rich	supple
Coffee + XJP7 (10 μg/mL)	brown	flowery	mellow	micro astringent	rich	supple
Coffee + HX1 (10 μg/mL)	brown	flowery	mellow	soft	rich	supple
Coffee + Super Red (10 μg/mL)	brown	flowery	moderate	soft	rich	supple
Coffee + Red Love (10 μg/mL)	brown	flowery	mellow	soft	rich	supple
Coffee + Fuji (10 μg/mL)	brown	flowery	moderate	soft	rich	supple
