# Supplementary material for: Red-Fleshed Apple Anthocyanin Extract Reduces Furan Content in Ground Coffee, Maillard Model System, and Not-from-Concentrate Apple Juice
Source: Foods. 2021 Oct 13;10(10):2423. doi: 10.3390/foods10102423 (PMC8535902; doi:10.3390/foods10102423)
Supplement: Supplementary file 1 [file foods-10-02423-s001.zip › foods-1386660-supplementary/Supplementary Materials/Supplementary Materials- Table S4.pdf]

Table S4. Sensory assessment of NFC apple juice after adding RAAE.

Group	Color	Aroma	Sweetness and Sourness	Abundance	Astringency	Aftertaste	Global Impression
Fuji + XJ3 (20 μg/mL)	khaki	flat aroma	sweet-sour	moderate	soft	flat	supple
Fuli + XJ3 (20 μg/mL)	khaki	flat aroma	sweet	moderate	soft	flat	supple
Fuxing + XJ3 (20 μg/mL)	yellowish	fragrance	sour-sweet	moderate	soft	obvious	supple
2010-W17-1Z-N9 + XJ3 (20 μg/mL)	khaki	flat aroma	slightly sweet	moderate	micro astringent	flat	hard
2008-W18-N16 + XJ3 (20 μg/mL)	maize-yellow	fresh	tart	moderate	micro astringent	obvious	harmonious
2010-W14-11Z-N9 + XJ3 (20 μg/mL)	maize-yellow	flat aroma	aceti	moderate	soft	flat	round
2010-W14-12Z-N3 + XJ3 (20 μg/mL)	buff	flat aroma	aceti	moderate	soft	flat	supple
2010-W14-3Z-N3 + XJ3 (20 μg/mL)	maize-yellow	fresh	tart	moderate	micro astringent	obvious	hard
2010-W10-3Z-N2 + XJ3 (20 μg/mL)	yellowish	aromatic	sweet-sour	mellow	soft	obvious	full
2009-E11-S5 + XJ3 (20 μg/mL)	wheat	aromatic	aceti	moderate	soft	obvious	supple
2010-W5-N2 + XJ3 (20 μg/mL)	wheat	flat aroma	sweet-sour	moderate	soft	flat	supple
2010-W7-8Z-N8 + XJ3 (20 μg/mL)	maize-yellow	flat aroma	sweet-sour	mellow	soft	obvious	supple
