# Supplementary figures and images for: Red-Fleshed Apple Anthocyanin Extract Reduces Furan Content in Ground Coffee, Maillard Model System, and Not-from-Concentrate Apple Juice
Source: Foods. 2021 Oct 13;10(10):2423. doi: 10.3390/foods10102423 (PMC8535902; doi:10.3390/foods10102423)

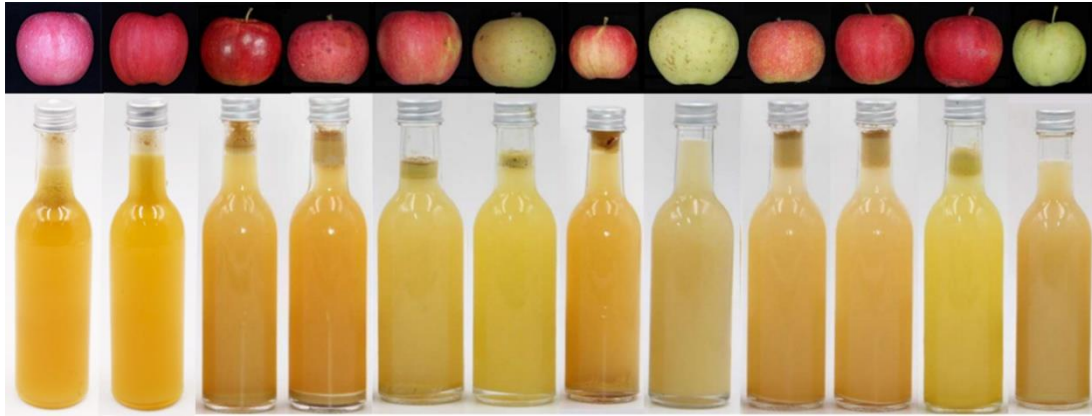

**Figure S2.** Appearance of fruits and juices of different apple varieties (strains).

Supplement: Supplementary file 1 [file foods-10-02423-s001.zip › foods-1386660-supplementary/Supplementary Materials/Supplementary Materials- Figure S2.pdf]
